# Development of a predictive model for failed extubation in patients with mechanical ventilation

**DOI:** 10.1186/2197-425X-3-S1-A1003

**Published:** 2015-10-01

**Authors:** J Sara, O Hernandez, F Jaimes

**Affiliations:** Clinica Medellín, Medellin, Colombia; Universidad de Antioquia, Medellin, Colombia; Instituto Neurológico de Colombia, Medellin, Colombia; Universidad de Antioquia, Internal Medicine, Medellin, Colombia

## Introduction

Patients with mechanical ventilation at the intensive care unit have failed extubation in 5 to 25% of the cases. There are many factors associated with failed extubation, but they have not been analyzed as a predictive model.

## Objectives

To develop a predictive model for failed extubation.

## Methods

A historical cohort study was conducted in a mixed intensive care unit with 40 beds at a University Centre. Patients analyzed were those admitted between 2010 and 2014, over the age of 16 years, with invasive mechanical ventilation for more than 24 hours and who overcome a spontaneous breath testing (SBT). Predictive variables: age, gender, days of intubation, length of intravenous sedation, cardiopulmonary diagnosis, BUN, creatinine, hemoglobin, PaO_2_/FIO_2_, PCO_2_, Glasgow scale, APACHE II, number of SBT, fluid balance 24 hours before extubation, cumulative fluids balance, positive culture of tracheal aspirate and use of inotropics before or during extubation. Failed extubation was defined as the need for invasive or non-invasive mechanical ventilation within the 48 hours after extubation. A multivariate logistic regression was fitted for predictive model selection, with assessment of discrimination by AUC-ROC and calibration by Hosmer-Lemeshow goodness-of-fit test.

## Results

From 5446 clinical records that were evaluated, 1017 entered the study and there was failed extubation in 157 (15.4%). A predictive model with the variables PaO_2_/FIO_2_, hemoglobin, accumulated fluid balance, cardiopulmonary diagnosis, APACHE II and BUN with good calibration (H-L goodness-of-fit = 0.579) and acceptable discrimination (AUC-ROC = 0.689) was obtained.

## Conclusions

A model using simple variables of routine measurement at ICU was predictive for failed extubation in our setting; it has good calibration and acceptable discrimination. This model needs to be validated in other intensive care units, and optimized considering different variables or definitions.

